# Discovering *Penicillium polinicum* with High-Lytic Capacity on *Helianthus tuberosus* Tubers: Oil-Based Preservation for Mold Management

**DOI:** 10.3390/plants10020413

**Published:** 2021-02-23

**Authors:** Abdulaziz A. Al-Askar, Ehsan M. Rashad, Khalid M. Ghoneem, Ashraf A. Mostafa, Fatimah O. Al-Otibi, WesamEldin I. A. Saber

**Affiliations:** 1 Botany and Microbiology Department, Faculty of Science, King Saud University, Riyadh 11451, Saudi Arabia; ashraf812@yahoo.com (A.A.M.); falotibi@ksu.edu.sa (F.O.A.-O.); 2 Seed Pathology Research Department, Plant Pathology Research Institute, Agricultural Research Center (ID: 60019332), Giza 12112, Egypt; ehsanrashad78@yahoo.com (E.M.R.); khalid_ghoneem@yahoo.com (K.M.G.); 3 Microbial Activity Unit, Microbiology Department, Soils, Water and Environment Research Institute, Agricultural Research Center (ID: 60019332), Giza 12112, Egypt

**Keywords:** caraway oil, clove oil, essential oils, inulinase, cellulase, xylanase, amylase, inulin, blue mold

## Abstract

During preservation, Jerusalem artichoke (JA) tubers are subjected to deterioration by mold fungi under storage, which signifies a serious problem. A new blue mold (*Penicillium polonium*) was recorded for the first time on JA tubers. Penicillium mold was isolated, identified (morphologically, and molecularly), and deposited in GenBank; (MW041259). The fungus has a multi-lytic capacity, facilitated by various enzymes capable of severely destroying the tuber components. An economic oil-based procedure was applied for preserving and retaining the nutritive value of JA tubers under storage conditions. Caraway and clove essential oils, at a concentration of 2%, were selected based on their strong antifungal actions. JA tubers were treated with individual oils under storage, kept between peat moss layers, and stored at room temperature. Tubers treated with both oils exhibited lower blue mold severity, sprouting and weight loss, and higher levels of carbohydrates, inulin, and protein contents accompanied by increased levels of defense-related phytochemicals (total phenols, peroxidase, and polyphenol oxidase). Caraway was superior, but the results endorse the use of both essential oils for the preservation of JA tubers at room temperature, as an economic and eco-safe storage technique against the new blue mold.

## 1. Introduction

Fruits and vegetables are consumed fresh. Improper postharvest handling causes rapid deteriorations in the quality of fresh produce and, in some cases, this results in products not reaching consumers at a satisfactory quality. The main cause of fruit deterioration is dehydration, with subsequent weight loss, color changes, softening, surface pitting, browning, loss of acidity, and microbial spoilage [1]. The majority of significant fruit losses result from decay caused by fungi such as *Penicillium* (green mold), *Botrytis, Monilia*, and others, leading to color changes and softening, accompanied by various economic losses that are dependent on the fruit type and storage conditions [2,3]. Moreover, the presence of *Penicillium* spp. may expose the health of the ultimate consumers to possible risk, since the fungus is usually linked to the production of mycotoxins, e.g., patulin; harmful metabolites which are detrimental to human health [4,5].

Jerusalem artichoke (*Helianthus tuberosus* L. family: *Asteraceae*) is a tuber-producing plant. In addition to being used as livestock fodder and in the production of biofuels, its tubers are consumed in human food and some functional-food ingredients because of their high nutritive value and polysaccharides contents, such as inulin, and oligofructose [6,7]. Under storage conditions, several fungi associated with rotted JA tubers have been reported, which vary in their disease potentiality and economic significance, e.g., *Sclerotium rolfsii*, *Sclerotinia sclerotiorum* [8,9,10], *Botrytis cinerea*, *Rhizopus stolonifera*, *Penicillium,* and *Fusarium* species [11], and *Rhizoctonia solani* [12].

In addition to the health hazards of manufactured fungicides, the development of pathogen resistance is another problem. The fungicides of biological origin, such as essential oils, representing a promising alternative approach not only for controlling postharvest decay but also for delaying the natural deterioration of stored fruits [13]. In general, such oils could be used as natural additives to extend shelf-life during the preservation of stored fruits. The levels of essential oils and their ingredients are determent factors in this respect [14]. Furthermore, essential oils represent ecofriendly, biodegradable, multifunctional, and nonpersistent natural products that reduce the risk of pathogen resistance build-up against chemicals because they contain two or more stereoisomers that target multi-sites on the pathogen’s cell membrane [9].

Essential oils, including clove (*Syzygium aromaticum* L. Merr. and Perr.) and caraway (*Carum carvi* L.), have been widely used in folk medicine as digestive, carminative, and lactogenic, antidiabetic, and analgesic therapeutic agents. Additionally, many other pharmacological aspects of such oils—importantly, their antioxidant, antiseptic, antibacterial, antifungal, antiviral, and insecticidal properties—have been reported [15].

**Figure 1:**
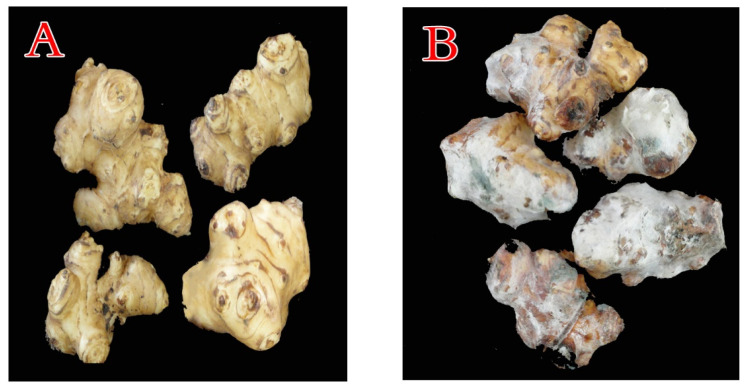
Tubers of Jerusalem artichoke (JA) stored under cooling conditions, showing the healthy (**A**) and blue mold decayed (**B**) tubers.

During 2019 (November up to March at Ismailia, Dakahlia, and Cairo provinces, Egypt) survey of post-harvest diseases of cool-stored JA tubers, a significant economic loss caused by blue mold was observed. The initial symptoms of blue mold include sections of—or the entire tuber tissue—displaying a soft, watery, discolored appearance, followed by the emergence of a dense white mycelium, which, with age, rapidly becomes covered with bluish spores (Figure 1). Finally, the whole tubers decompose and ferment. This process is accompanied by the production of bad odors. Such decayed samples were gathered, for further follow-up during the current study.

Therefore, this study was conducted to evaluate the in vitro antifungal activity of essential oils in the management of the phytopathogenic fungus: *Penicillium polonium*. This study also aimed to evaluate the application of the most potent essential oil as an economic alternative preservative to control the development of blue mold disease in JA tubers under storage conditions, as well as to enhance their storability and maintain their quality characteristics.

## 2. Materials and Methods

### 2.1. Isolation of Blue Mold

An isolation trial was conducted from 15 decayed samples of JA tubers, stored under cool conditions. Blue moldy JA tubers were washed with sterilized tap water and cut into sections with a sterilized scalpel. Fifty pieces (approximately 1 cm long) of each sample were surface sterilized in 1% NaOCl for 3 min and 70% ethanol for 20 s and then rinsed with several changes of sterile distilled water. Ten pieces of the sterilized tubers were plated out on potato dextrose agar medium (PDA) supplemented with an antibacterial agent (0.1 mg/l L-chloramphenicol and 0.3 mg/l streptomycin sulfate). The plated Petri dishes were then incubated at 25 ± 2 °C for 5 days under dark conditions. The recovered fungi were detected by examination of each JA piece under a stereoscopic binocular microscope (6–50×). The fungal habit characters were obtained and a light microscope was used for confirmation.

#### 2.1.1. Fungal Purification and Identification

Samples that showed the typical characteristics and symptoms of blue mold were selected, on which, three isolates (ARS20, ARS21, and ARS22) were detected. The emerged fungal growth was picked and purified using the hyphal tip and/or single spore techniques, before being transferred to plates containing PDA in the presence of the same antibiotic (0.1 mg/L L-chloramphenicol and 0.3 mg/l streptomycin sulfate). For studying the morphological features of the isolates, a two-point inoculation on the media of PDA, Czapek dox agar (CDA), and malt extract agar (MEA), using a dense conidial suspension was conducted, and then this was incubated at 25 ± 2 °C for 7 days. Pure cultures of developing fungi were transferred to slants containing potato carrot agar medium and kept at 4 °C for further studies. The occurrence percentage for each of the three isolates in the different samples was recorded.

Fungal culture characteristics, including size, texture, colony appearance, and surface pigmentation were investigated and identified [16,17]. For the determination of morphological structures, portions of fungal growth were mounted in lactophenol cotton blue stain on clean slides. The prepared slides were examined under a light microscope using the 40× and 100× objectives for identification of vegetative mycelium; septation, diameters, conidiophores, and the reproductive structures.

#### 2.1.2. Pathogenicity Test

The three isolates of *Penicillium polonicum* (ARS20, ARS21, and ARS22) were subjected to the pathogenicity test, which was conducted to determine the decaying potential of the fungal isolates. Healthy-appearance tubers of Fuseau cultivar, obtained from the Vegetable Research Department, Horticulture Research Institute, Agricultural Research Center, Giza, Egypt, were washed and surface sterilized in 1% NaOCl for 3 min, then washed twice with sterile distilled water and left to dry. Inoculation was carried out under sterilized conditions by making a small hole in the tuber using a sterile 5 mm cork-borer and inserting a 5 mm disc of a 5-day-old culture of the tested fungus in the hole. Control tubers were treated in the same manner, except that an uninoculated disk of PDA was used. Inoculated tubers were then stored individually in sterile humid plastic boxes at 25 °C. Over 10 days, the artificially infected tubers with the three pathogenic isolates were investigated for rotting potentiality. Twenty tubers were used for each isolate.

The severity (%) of the tuber rot was evaluated based on a scale from 0 to 9, with some modification; where: (0) no symptoms of rot, (1) up to 10% of tuber rot, (2) more than 10 up to 20% of tuber rot, (3) more than 20 up to 30% of tuber rot, (4) more than 30 up to 40% of tuber rot, (5) more than 40 up to 50% of tuber rot, (6) more than 50 up to 60% of tuber rot, (7) more than 60 up to 70% of tuber rot, (8) more than 70 up to 80% of tuber rot, and (9) more than 80% of tuber rot [9]. The severity% (*S*) was then calculated using the following formula:

$$S=\frac{\sum n}{N\times 9}\times 100$$

where; ∑*n* is the sum of the individual ratings; *N* = total number of JA tubers assessed and 9 is the highest score on the severity scale.

#### 2.1.3. Molecular Identification

The selected *P. polonicum* ARS20 isolate was identified molecularly, by applying the molecular biological procedure of DNA isolation and amplification via the polymerase chain reaction (PCR). DNA isolation and purification were performed using DNeasy Tissue Kits (QIAGEN-Germany), followed by determination of DNA concentration through comparing with standard lambda DNA on 1% (*w*/*v*) agarose gel. Samples were then diluted to 20 ng DNA µL and kept at −20 °C.

For PCR amplification and sequencing of the internal transcribed spacer (ITS), the reaction mixture consisted of 1× buffer (Promega), 1.5 mM MgCl2, 1U Taq DNA polymerase (GoTaq, Promega), 0.2 mM dNTPs, 30-picomole of each primer (ITS5-F (5′-GGAAGTAAAAGTCGTAACAAGG-3′) and ITS4-R (5′-TCCTCCGCTTATTGATATGC-3′)), and 30 ng of genomic DNA, and ultra-pure water was added to a final total volume of 50 µL.

Thermo-cycling PCR amplification was performed by Perkin-Elmer/GeneAmp^®^ PCR System 9700 (PE Applied Biosystems, Foster City, CA, USA) programmed to fulfill 40 cycles (30 s each) after an initial denaturation cycle for 5 min at 94 °C. The annealing step was conducted at 45 °C for 30 s, and the elongation step was conducted at 72 °C for 30 s. The primer extension segment was extended to 7 min at 72 °C in the final cycle. Purification was accomplished to eliminate unincorporated PCR primers and dNTPs from PCR products using the Montage PCR Clean-up kit (Millipore).

For nucleotide sequence analysis, similar sequences were searched using the NCBI BLASTn online tool http://ncbi.nlm.nih.gov/BLAST/ (accessed on 1 December 2020) and aligned using Align Sequences Nucleotide BLAST with the nucleotide collection (nr/nt) database.

A phylogenetic tree was constructed based on the ITS region sequence comparisons of length polymorphism of the amplified PCR, and sequences from the database using blast tree and aligned using aligned sequences nucleotide BLAST. The phylogeny was tested using the bootstrap method with 2000 replications and generated based on the UPGMA statistical method. The software package; MEGA 10 was used for multiple alignments and phylogenetic analysis based on the neighbor-joining method. The obtained sequence (688 bp) was deposited in GenBank to obtain the closely related fungi sequences; then, the accession number of the fungal strain was received.

### 2.2. Extraction and GC Analysis of the Essential Oils

Essential oils of caraway seeds (*Carum Carvi* L.), clove flower buds (*Eugenia carypylata* L.), stems and leaves of sweet wormwood (*Artemisa annua* L.) and spearmint leaves (*Mentha spicata* L.) were extracted separately from 200 g samples. Extraction was done through a hydro-distillation procedure of the plant materials for 3 h using a Clevenger apparatus [18]. The extracted pure essential oils were stored at 4 °C in clean amber dark glass bottles until used.

Based on the antifungal screening test, caraway and clove oils—the most effective against the growth of the fungal pathogen—were analyzed by a Gas Chromatography-Flame Ionization Detector (FID), using a DsChrom 6200 gas chromatograph equipped with a flame ionization detector and a column of BPX-5, 5% phenyl (Equiv.) polysillphenylene-siloxane 30 m × 0.25 mm ID × 0.25µm film with a sample size of 1 μL. The temperature program ramp was increased at a rate of 10 °C per min from 70 to 200 °C. The detector temperature, FID, was 280 °C. The carrier gas was at a flow rate of (mL/min) 30 (N_2_):30 (H_2_):300 (air). The main compounds of the essential oils were identified by matching their retention times with those of the standard authentic samples injected under the same conditions. The relative percentage of every compound was calculated from the area of the peak corresponding to each compound.

### 2.3. Anti-P. polonicum Activity of the Essential Oils

For the in-vitro assessment of the activity of the essential oils against *P. polonicum*, initially, each of the four essential oils was screened and evaluated. Erlenmeyer flasks (250 mL), containing 50 mL sterilized potato dextrose (PD) broth medium and 0.5% Tween-20 were prepared. After sterilization, each essential oil was separately added to flasks to cover a range of concentrations from 1 to 4% (*v*/*v*). Flasks were inoculated using 1 mL of spore suspension (prepared from 5-days old culture grown on plates of PD agar at 28 ± 2 °C for 5 days and suspended in sterile saline (0.85%) to adjust the count to 106 spores/mL). Then, the flasks were incubated in the dark at 28 ± 2 °C for 7 days. An untreated medium was used as a control. Triplicate flasks were used for each treatment. At the end of the incubation period, the mycelial mats were harvested, washed several times with distilled water, and oven-dried to a constant weight at 70 °C. The biological activity of oils was expressed as the growth percentage in the mycelial dry weight, in relation to the control (0% oil).

### 2.4. Enzymatic Profile of P. polonicum ARS20

#### 2.4.1. Fermentation Conditions

To explore the enzymatic profile of *P. polonicum* in the presence of the most effective oils (caraway and clove), another fermentation trial was performed. The fermentation procedure and the medium of [19] were used with some modifications. Erlenmeyer flasks containing 50 mL of the medium were supplemented with one gram of ground cold-dried powder of *H. tuberosus* tubers. After sterilization, the medium was further supplemented with both oils at concentrations of 0.5–2.5%. Then, the medium was inoculated with 1 mL of the previously-prepared spore suspension. The batch fermentation was carried out in the dark under shaking conditions (200 rpm) at 28 ± 2 °C for 7 days. After incubation, the mycelium was removed by centrifugation (5000 rpm for 20 min) and the culture filtrate was assayed for hydrolytic enzymes.

#### 2.4.2. Assay of Enzymes

Assays of filter paperase (FPase) and xylanase in the post-culture filtrate were performed using microcrystalline cellulose [20], and xylan [21], respectively, as substrates. The substrates (0.5%) were individually dissolved in citrate buffer (0.05 M, pH 4.8), the reaction mixture (1 mL of the filtrate and 1 mL substrate-buffer solution) was incubated at 50 °C for 60, and 30 min, respectively.

The activity of α-amylase was assayed in a reaction mixture containing amylase and 0.5% soluble starch in phosphate buffer (pH 6.5), incubated at 30 °C for 10 min [22].

The inulinase and invertase activities were assayed in reaction mixtures containing 1 mL of 0. 5% inulin or 50 mM sucrose in 0.2 M sodium acetate buffer (pH 4.8), plus 1 mL of the enzyme solution. Incubation was performed at 50 °C for 20 min. [19].

Polygalacturonases (PGase) activity was assayed in a reaction mixture composed of the fungal filtrate and 0.1 M sodium acetate buffer (pH 5.2), after 30 min incubation at 40 °C, using the 3,5-dinitrosalicylic acid (DNSA) method with D-galacturonic acid monohydrate as the standard [23].

The released reducing groups by the previous enzymatic action were determined by the DNSA method [24]. With the aid of standard curves, the enzyme unit (U) was defined as the amount of enzyme required to release 1 µmol/mL/min of reducing-units of glucose (FPase and amylase), xylose (xylanase), an equimolar solution of glucose and fructose (inulinase and invertase), and polygalacturonic acid (PGase), under the test conditions.

The proteolytic activity (protease) was quantitatively assayed in a reaction mixture of crude extract and casein as a substrate. After incubation at 37 °C for 10 min, free amino acids were separated by trichloroacetic acid [25], the released amino acids were measured at A280. One U of protease activity was defined as the amount of the enzyme resulting in the release of 1 µg of tyrosine equivalent/mL/min under the assay conditions.

### 2.5. Essential Oils vs. Blue Mold under Storage

Healthy JA tubers were washed by water, surface disinfected in 1% NaOCl for 3 min, then rinsed in sterilized water and left to dry. Meanwhile, a 2% concentration of the selected essential oils (clove and caraway) was prepared. The inoculum of *P. polonicum* was scaled up in a 250 mL Erlenmeyer flask containing 50 mL of sterilized PD-broth medium inoculated with 1 mL (10^6^ spores/mL), then incubated in the dark at 28 ± 2 °C for 10 days. The mycelial mat was then harvested and washed gently with sterile distilled water. Twenty grams of the fungal mat were mixed thoroughly and blended with distilled water (one liter). The resultant homogenized suspension was adjusted at 10^6^ cfu/mL.

Six groups of tubers (15 kg each) were separated into (i) untreated control (C); (ii) untreated and infected by *P. polonicum* (ARS20) pathogen (P); (iii) treated with caraway oil (CR); (iv) treated with clove oil (CL); (v) treated with caraway oil plus infection by *P. polonicum* (CRP), and (vi) treated with clove oil plus infection by *P. polonicum* (CLP). For oil treatments, tubers were soaked individually in both essential oils for 20 min, then left for about 2 h. In case of artificial infection, the tubers were sprayed with the fungal suspension of *P. polonicum* until run-off. The tubers were then stored at 25 ± 2 °C in plastic boxes (40 × 20 × 20 cm) within layers of moistened peat moss (FLAGMA, EcoPeatMix, LLC, Saint-Petersburg, RU, Peatmoss Neutralized, pH 5.5–6.5, Fraction: 0–10 mm and relative humidity 55–65%) at a rate of 1:1 (JA tubers: peat moss).

Storage lasted for 4 months, meanwhile, tuber samples were withdrawn, regularly, at one-month intervals, to follow up the changes under the proposed storage conditions. The disease index of blue mold (tuber rot severity, %) was measured based on the previously mentioned scale [9]. The tubers were further assessed for defense-related enzymes, i.e., peroxidase and polyphenol oxidase [26], and total phenols [27]. The tubers were analyzed for inulin [28], total carbohydrate [29], and protein [30] contents. The percentage of sprouting, weight losses, and dry matter of tubers were also measured.

### 2.6. Statistical Analysis

All the experimental data are presented as means ± standard deviation (±SD). At least six replicates were used.

## 3. Results

### 3.1. The Blue Mold

#### 3.1.1. Isolation, Morphological, and Microscopic Identification

**Figure 2:**
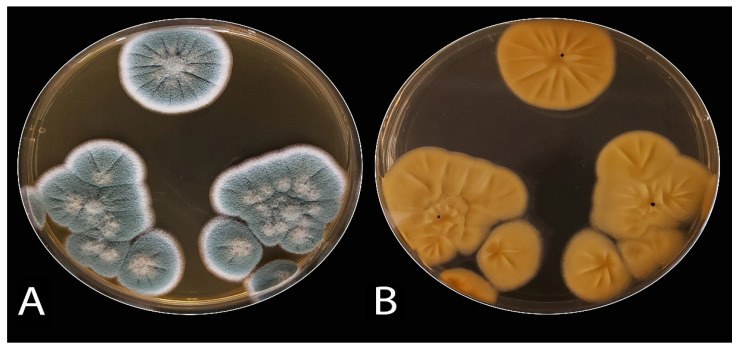
Colony morphology of *P. polonicum* on malt extract agar (MEA) medium: (**A**) obverse and (**B**), reverse.

**Figure 3:**
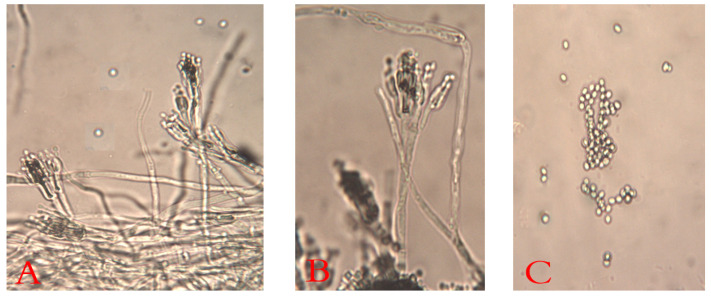
Microscopic micrograph of *P. polonicum* shows the typical terverticllate conidiophores ((**A**,**B**), at ×400) with smooth septate stripes and the spherical conidia ((**C**), at ×400).

The decayed JA tubers stored under cooling conditions and with the typical symptoms of blue mold were selected. They were mostly specified as belonging to *Penicillium* spp. The Penicillium isolates (ARS20, ARS21, and ARS22) were subjected to further characterization. The initial morphological investigation on PDA, CDA, and MEA media at 25 ± 2 °C after 7 days showed blue–green, velutinous on MEA. Colonies for the three isolates have a distinct reverse, often with a yellow–brown color (Figure 2). The mean colony diameters (mm) for ARS20, ARS21 and ARS22 were 28.9 ± 0.96, 27.6 ± 0.50 and 29.8 ± 0.65 (on PDA), 31.56 ± 0.53, 30.6 ± 0.75, and 30.97 ± 0.6 (on MEA) and 28.56 ± 0.53, 29.21 ± 0.42, and 30.10 ± 0.4 (on CDA), respectively. The microscopic examination of the colonies revealed two-stage branched conidiophores, adpressed phialides with rough-walled stipes, and conidia that were smooth, globose to sub-globose, and borne in columns. Conidial dimensions for ARS20 were 3.0–3.6 (3.15) × 2.6–3.4 (2.9) μm, for ARS21 were 2.85–3.35 (3.1) × 2.57–3.55 (3.0) and for ARS22 were 2.95–3.8 (3.32) × 2.7–3.65 (3.16) μm in diameter (Figure 3). The three isolates displayed the typical characteristics of *P. polonicum* K. Zaleski.

#### 3.1.2. Pathogenicity Potential of the Blue Mold

**Figure 4:**
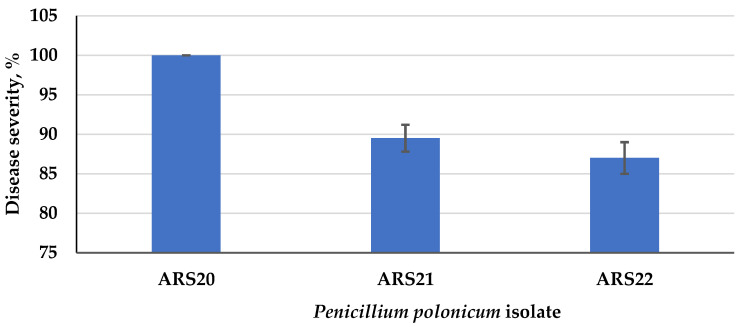
Disease severity of the three tested isolates of *Penicillium polonicum* on JA tubers.

**Figure 5:**
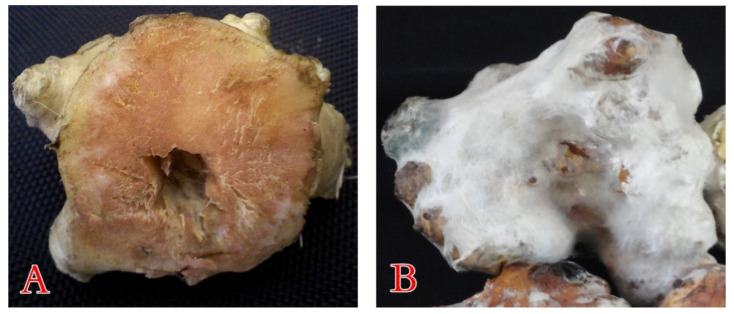
Artificially infected JA tubers with *P. polonicum* ARS20, showing the healthy (**A**) and the typical symptoms of blue mold (**B**) on tubers.

For the selection of the most severe blue mold isolate, a pathogenicity test was carried out on the three *P. polonicum* isolates. All the isolates developed the typical symptoms of blue mold on the artificially infected tubers, while the non-inoculated control remained symptomless. Among the tested blue mold isolates, *P. polonicum* ARS20 was the only fungal pathogen that recorded 100% disease severity at 25 °C (Figure 4). It was easily recognized by the presence of water leaching on the tuber after 5 days, later associated with a dense growth of white mycelium, and blue–gray mold spread rapidly, covering the entire tuber surface after about 10 days (Figure 5). The other two *P. polonicum* isolates, ARS21 and ARS22, came next, with disease severities of 89.5 and 87.0%, respectively. To our knowledge, this is the first report of a *P. polonicum* pathogen on stored JA tubers in Egypt. The Koch’s postulates were considered in order to ensure the ability of various *P. polonicum* isolates to develop the same disease symptoms on tubers.

#### 3.1.3. Molecular Identification

**Figure 6:**
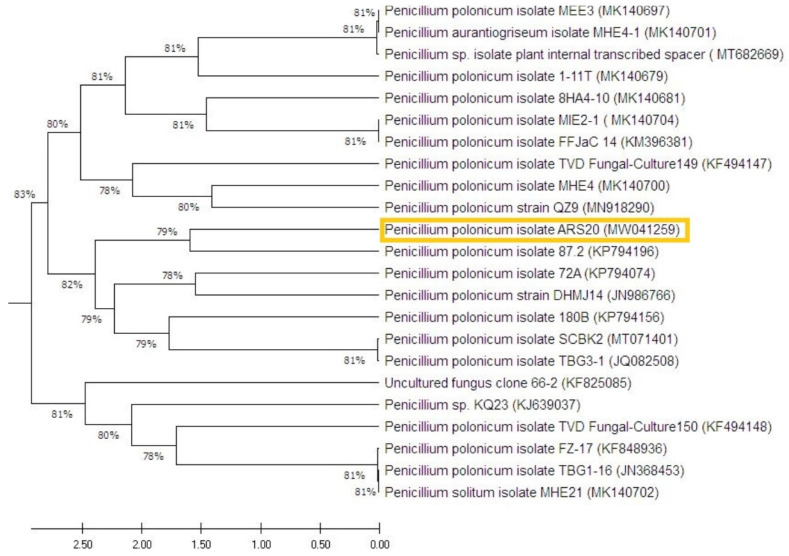
Molecular phylogenetic tree of the partial sequence of the internal transcribed spacer, showing the position (highlighted with the yellow rectangle) of *Pencillium polonicum* ARS20 (MW041259) with respect to the closely related sequences obtained from GenBank.

The selected fungal strain (*P. polonicum* ARS20) was characterized by the molecular technique of ITS as the perfect tool for identification. This technique compares the sequence that codes for the 18S rRNA gene after PCR amplification. From the Blast analysis, the strain ARS20 displayed high similarity with the formerly identified *P. polonicum* on the GenBank-constructed phylogenetic tree of the RS20 isolate (Figure 6). This is in line with previous morphological identifications. The GenBank accession number of the present fungal strain *P. polonicum* ARS20 is MW041259.

### 3.2. Essential Oils vs. P. polonicum

#### 3.2.1. Essential Oils vs. Fungal Growth

**Figure 7:**
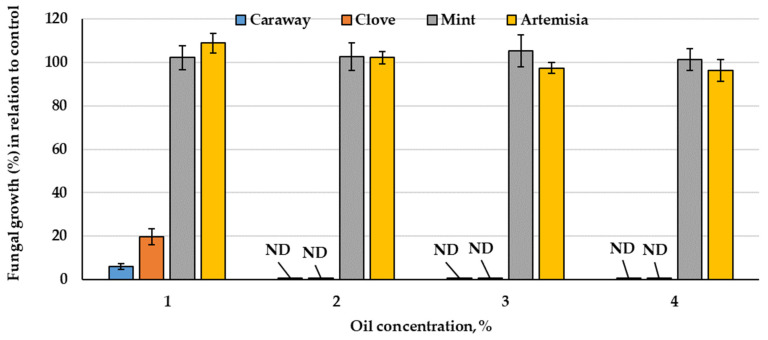
The growth of *P. polonicum* (%) as a response to various essential oils concentrations, in relation to control. ND: not determined due to the absence of fungal growth.

The comparative response of *P. polonicum* growth in response to four tested oils was screened. The results of fungal growth percent in relation to the control (100%) show (Figure 7) a large variation in *P. polonicum* growth due to the kind of oil tested. Two diverse patterns could be noticed, mint and artemisia oils induced fungal growth up to 2% of the oil. Then, opposite to artemisia oil that slightly reduced growth at 3 and 4%, mint oil continued inducing the growth (>100%) at higher concentrations (4%). From point of view of effectiveness and economics, both oils were omitted from the subsequent trials. Another pattern was shown by the other two oils, which exerted a declining pattern of fungal growth. Both caraway and clove oils vigorously reduced growth at 1%, followed by full inhibition at the higher concentrations (2% and above). The latter two oils were further explored in relation to the biocatalytic activity of the fungus.

#### 3.2.2. GC Analysis of Caraway and Clove Oils

**Figure 8:**
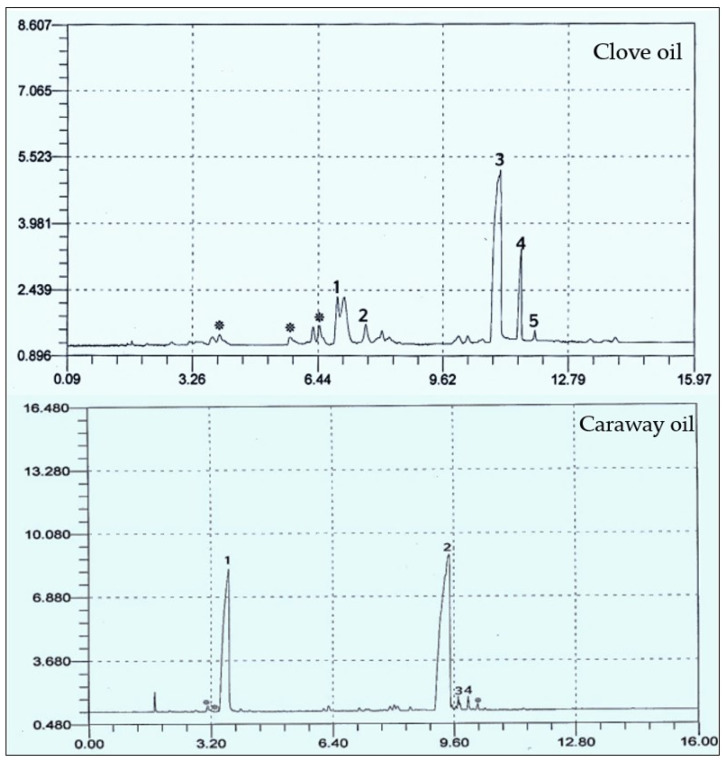
Chromatographic analysis of clove and caraway essential oils, showing the peaks of various components. For clove oil, (1) methyl salicylate, (2) bicyclobutylidiene, (3) eugenol, (4) eugenyl acetate, and (5) farnesene. For caraway oil, (1) limonene, (2) carvone, (3) perilla alcohol, and (4) carveol. Peaks designated with astricts (*) are unidentified compounds.

After extraction, the composition of the most two effective oils was chemically screened using GC (Table 1 and Figure 8). The major components in clove and caraway oils were eugenol and carvone, respectively. Caraway oil is mainly constituted of monoterpenes, while the main component in clove oil, i.e., eugenol, is a phenylpropanoid. However, eight compounds were identified in clove essential oil, i.e., methyl salicylate, bicyclobutylidiene, eugenol, eugenyl acetate, and farnesene, in addition to three unidentified compounds. The major compounds were eugenol (49.077%), methyl salicylate (19.274%), and eugenyl acetate (11.551%). Of the seven compounds separated in caraway oil, four were identified, which are carvone (60.398%), limonene (35.265%), perilla alcohol (1.430%), and carveol (1.106%).

### 3.3. Enzymatic Profile of P. polonicum under Oil Stress

The enzymatic system of *P. polonicum* was assayed to discover the pattern and mode of action of the hydrolysis capacity of the fungus in the presence and absence of essential oils (Table 2). The secretion of the fungal enzymes showed a declining pattern as the concentrations of both oils were increased. The response varied according to the enzyme, for example, FPase disappeared at 1% (clove) and 1.5% (caraway), despite this, the fungus still demonstrated some ability to grow at these concentrations. Furthermore, the fungus failed to show any xylanolytic activity, even at the lowest caraway concentration. The other enzymes (amylase, inulinase, invertase PGase, and protease) displayed various responses in their activities at 1.5% concentrations of both oils. At a 2% concentration of both oils, the whole enzymatic system was inhibited and no growth was detected.

### 3.4. Essential Oils against P. polonicum under Storage

#### 3.4.1. Disease Severity

**Figure 9:**
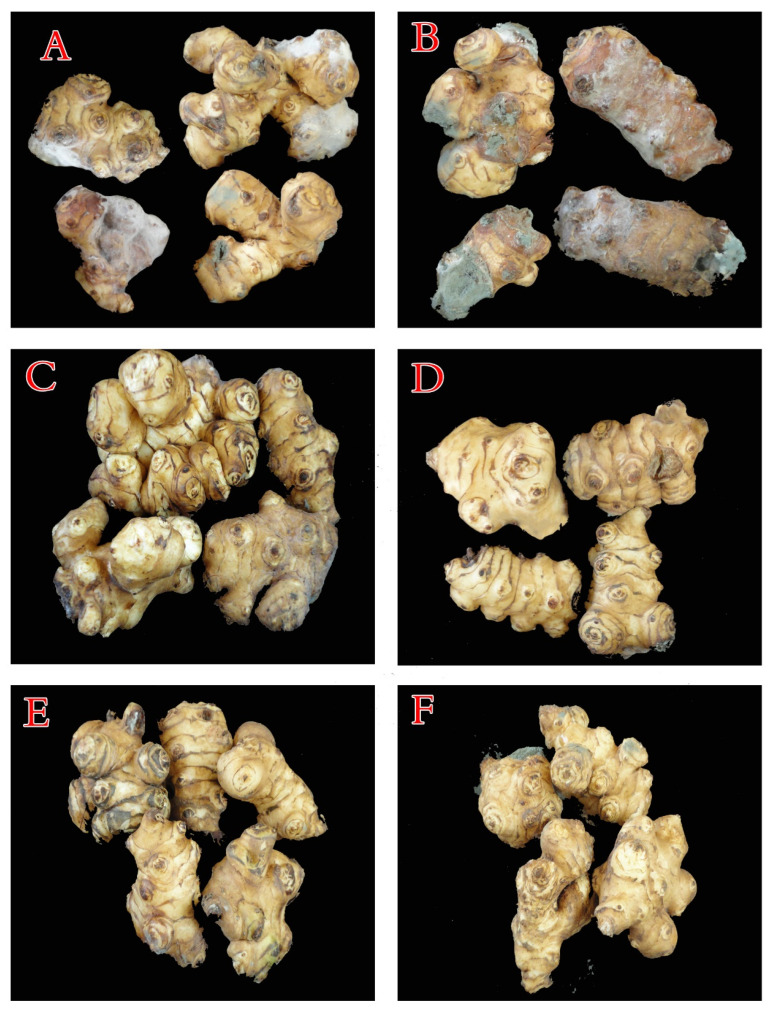
Effect of clove and caraway essential oils on control of JA tuber decay caused by *P. polonicum* under storage conditions at 25 °C after 2 months. (**A**) = untreated control (C), (**B**) = untreated and infected by *P. polonicum* (ARS20), (**C**) = treated with caraway oil (CR), (**D**) = treated with clove oil (CL), (**E**) = treated with caraway oil plus infection by *P. polonicum* (CRP) and (**F**) = treated with clove oil plus infection by *P. polonicum* (CLP).

The infected JA tubers were treated with caraway and clove oils, and their impact on the blue mold—caused by *P. polonicum* ARS20 development—was evaluated under storage conditions (Table 3). Under infection stress, a positive relationship was detected between the disease severity and the progress of the storage period. In this respect, the treatment of the infected tubers with CRP prohibited the appearance of disease symptoms caused by the tested pathogen. JA tubers treated with CLP came next in this respect, as compared to the untreated-infected control. Without artificial infection stress, storage JA tubers treated with CR or CL, completely prevented the appearance of any infection symptoms throughout the 4-month storage period, after which, the tubers had fully deteriorated. After two months of storage, there were dramatic variations regarding the development of the pathogen on the contaminated stored JA tubers (Figure 9).

#### 3.4.2. Defense-Related Phytochemicals

**Figure 10:**
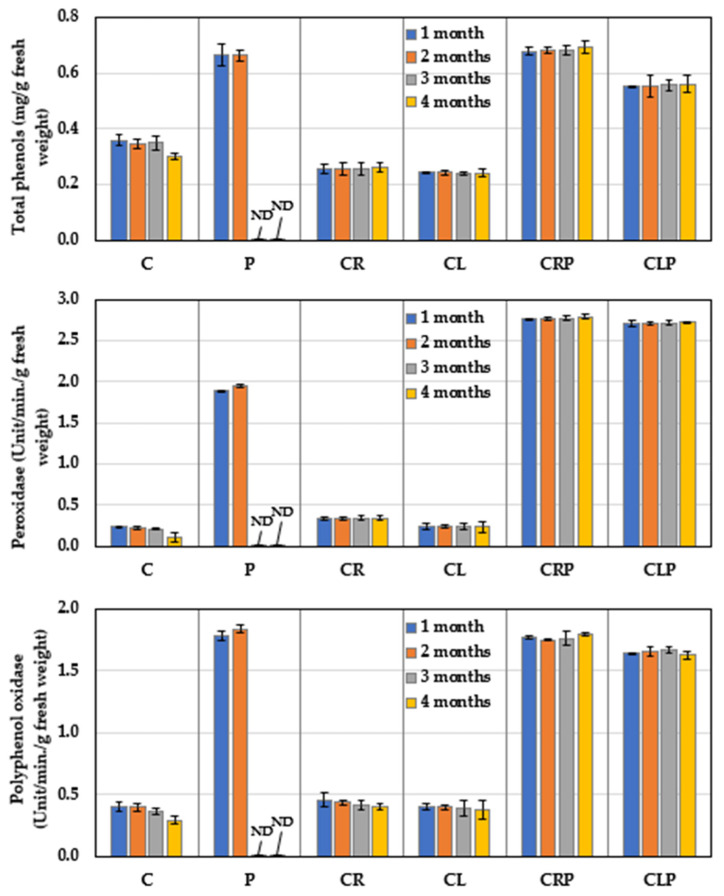
Defense-related phytochemicals of JA tubers treated with essential oils and infected with *P. polonicum* under storage at 25 ± 2 °C (mean ± SD). C: untreated control; P: untreated infection by *P. polonicum* (ARS20); CR: treated with caraway oil; CL: treated with clove oil; CRP: treated with caraway oil plus infection by *P. polonicum*; CLP: treated with clove oil plus infection by *P. polonicum*; ND: not determined due to full deterioration.

The comparative response of defense-related phytochemicals (peroxidase, polyphenol oxidase, and total phenols) of the stored JA tubers to CR or CL essential oils were measured over 4 months (Figure 10), in the presence or absence of *P. polonicum*. The obtained results indicate that JA tubers infected with *P. polonicum* showed a significant increase in total phenol levels and the activity of both enzymes, as compared to those of the untreated control and healthy JA tubers treated with either of the essential oils. On the contrary, under infection stress, treatment with the CR essential oil led to the highest increase in both enzymes, and phenol content. JA treated with Cl oil came next, ranking second when compared with untreated-infected tubers.

#### 3.4.3. The Structural Features of JA Tubers

**Figure 11:**
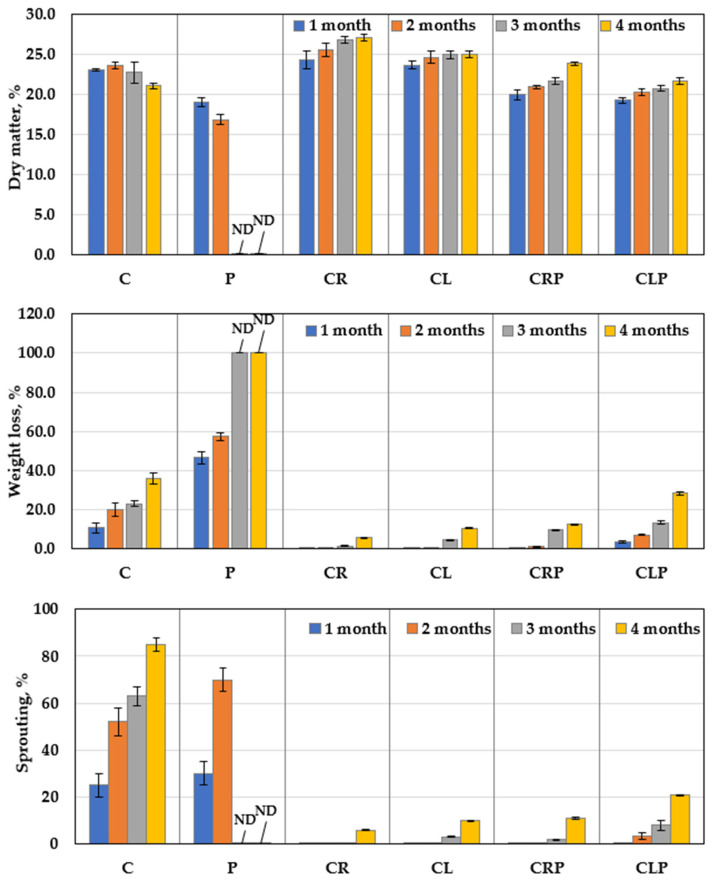
Comparative response features of JA tubers treated with essential oils after infection with *P. polonicum* under storage at 25 ± 2 °C (mean ± SD). C: untreated control; P: untreated infection by *P. polonicum* (ARS20); CR: treated with caraway oil; CL: treated with clove oil; CRP: treated with caraway oil plus infection by *P. polonicum*; CLP: treated with clove oil plus infection by *P. polonicum*; ND: not determined due to full deterioration.

The structural characteristics (dry matter, sprouting, and weight loss) of JA tubers as a response to CR and CL essential oils were measured (Figure 11) under storage conditions, either in the presence or absence of *P. polonicum* infection, as compared to the negative and untreated-infected control. The results revealed that the capability of CR and CL treatments to strongly reduce both sproutings and weight loss, in addition to a gradual increase in dry matter weight throughout the storage period being recorded, even in the presence or absence of the pathogen. Contrarily, over two months’ storage, the presence of the pathogen caused a pronounced increase in sprouting and reduction in dry matter in the infected control, accompanied by a gradual increase in weight loss.

#### 3.4.4. Chemical Content of JA Tubers

**Figure 12:**
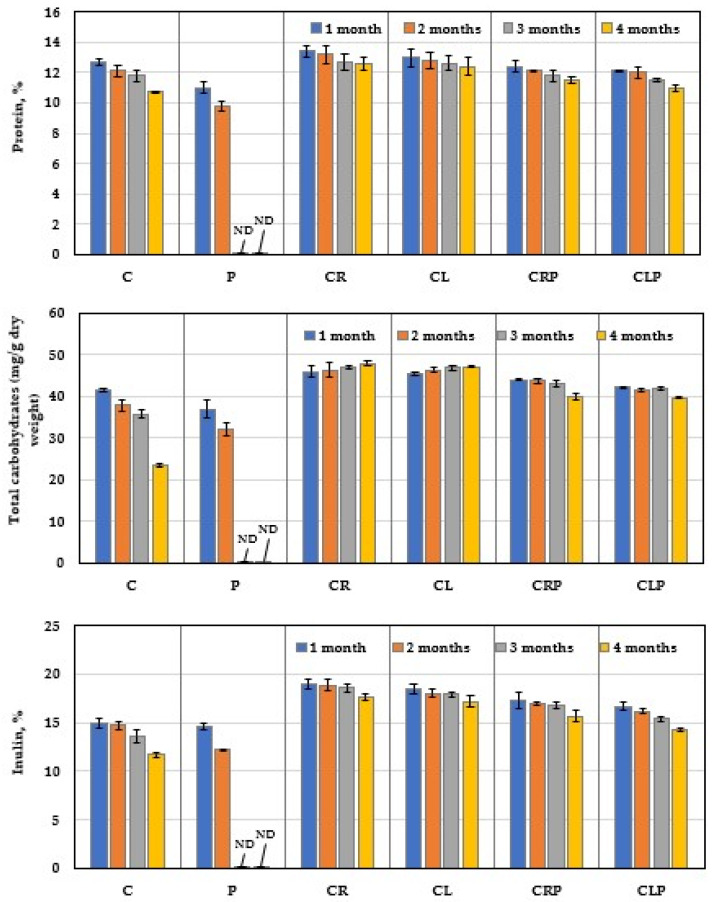
Comparative carbohydrates, inulin, and protein contents of JA tubers treated with caraway and clove essential oils and infected with *P. polonicum* under storage at 25 ± 2 °C (mean ± SD). C: untreated control; P: untreated infection by *P. polonicum* (ARS20); CR: treated with caraway oil; CL: treated with clove oil; CRP: treated with caraway oil plus infection by *P. polonicum*; CLP: treated with clove oil plus infection by *P. polonicum*; ND: not determined due to full deterioration.

Evaluation of the effect of CR and CL oils on protein, carbohydrates, and inulin contents of stored JA tubers in the presence or absence of *P. polonicum* stress infection was detected (Figure 12). Over the 2 months of storage, the untreated-infected treatment showed a fast degradation of protein, carbohydrates, and inulin contents, which positively correlated with the progress of the storage period.

A similar negative relationship, with a slow degradation, was detected between the untreated-control (C) and the progress of the storage period up to 4 months. Treatment of the uninfected tubers with CR and CL oils significantly reduced the degradation of their inulin, protein, and increase carbohydrate contents compared with the untreated-control, over 4 months of storage. Under infection stress, JA tubers treated with CR or CR oils effectively lowered the reduction in carbohydrate, inulin, and protein degradation compared with the untreated-infected control over the storage period.

## 4. Discussion

Isolation of the blue mold pathogen was performed, applying Koch’s postulates to ensure the precise determination of the causal microorganism. Following isolation, analysis of the morphological and microscopic features represents the first and most basic route for fungal identification. Investigation of the three blue mold isolates showed that their characteristics were in accordance with *P. polonicum* [17,31]. Penicillium is one of the largest and most important genera of microscopic fungi, with over 400 described species distributed worldwide. Its name comes from the Latin “penicillus”, which refers to the brush-like appearance of the conidiophores that resemble a painter’s brush [32]. Subsequent to visual identification, molecular identification methods were used.

Regarding the pathogenicity test, the three isolates of *P. polonicum* exhibited various degrees of disease severity. Isolates recovered from the artificially inoculated tubers showed the same morphological characteristics as those that previously appeared in the case of natural infection under storage that comes in agreement with Koch’s postulates. To our knowledge, this is the first report of *P. polonicum* on stored JA tubers. Previously, *Penicillium* spp. were reported as the most form of common storage rot pathogen, but specific species were not previously identified [8,11]. Confirmatory, *P. polonicum* has previously been reported as a psychro-tolerant xerophilic fungus that is associated with the spoilage of many foods and food products, such as peanuts, cereals, yam tubers, kiwifruit fruits, onions, and green table olives [17,31,33,34,35].

In order to ensure accuracy and confirmation, molecular identification is usually performed. This is highly sensitive and specific, with it being largely applied for the rapid identification of various fungi. The well-identified sequence—used for phylogeny creation—is fully annotated and shows a firm correlation with the other comparable fungal strains in the GeneBank database. Both ITS4 and ITS5 primers were used to amplify the nucleotide sequencing of the ITS region. This fragment region is uniform in a wide variety of fungal groups. This, in turn, aids in revealing the interspecific and, in some situations, intraspecific variation among organisms [36]. Furthermore, the sequences of this non-functional region are often extremely variable among fungal species [37], which is why such a region can be sufficient for fungal identification at the species level [38]. Technically, the repeated or multi-copy feature of the rDNA makes the ITS regions easy to amplify from a tiny sample of DNA [37]. Therefore, nucleotide sequencing of the ITS region is considered to provide quick and highly precise identification results compared to most other markers. Additionally, this can be used for barcode identification of a very broad group of fungi [39]. Interestingly, the molecular identification of *P. polonicum* ARS20 is in harmony with the morphological identification, with both identification methods confirming that the isolated blue mold (ARS20) is *P. polonicum*.

Clove and caraway oils showed a gradual reduction in fungal growth with increasing concentrations of both oils. Oils are known to inhibit fungal growth. The antifungal activity of caraway essential oil may be attributed to various antifungal phytochemicals that constitute a large fraction of the oil, such as carvone, limonene, carveol, pinen, and thujone [9]. The antagonistic feature of clove oil can be attributed to the presence of an aromatic nucleus and phenolic OH-group that can react with the phospholipids of the cell membrane, changing their permeability features [10]. Essential oils could be used as natural additives in the food industry, without the fear of potential health hazards, as well as in postharvest treatment to increase shelf-life due to their antifungal, antioxidant, antibacterial, and anti-carcinogenic properties [14].

The GC analysis revealed various components of the clove and caraway essential oils, with fractions showing evident antifungal capacity, which varied according to the distribution of the components of each fraction. The GC analysis showed an increase in the proportion of oxygenated compounds in caraway fractions in comparison to hydrocarbons, therefore, caraway oil could be considered to be highly oxygenated while clove oil is moderately oxygenated. Previously, oxygenated compounds have been reported to have bioactive inhibitory effects against several fungal strains, including numerous *Penicillium* strains, such activity of the oil could be attributed to the oxygenated carvone that possessed higher antifungal activity, as well as antioxidant activity [40,41,42]. Such compounds could also inhibit the rate of primary and the secondary oxidation product formation. Furthermore, the physical nature of the rich oxygenated essential oils, for example, their low molecular weight combined with a lipophilic character, allow them to penetrate the cell membrane more quickly compared to other substances [41,43]. Clove oil also showed good potential in inhibiting the growth of *Penicillium polonium*. The hydroxyl group on eugenol (the major component of clove oil) is thought to bind to cellular proteins, preventing the catalytic action of various fungal enzymes [44]

Contrarily to caraway and clove oils, mint and artemisia supported fungal growth at the initial concentration but slightly reduced growth with the advance of concentration levels. Mint slightly encouraged fungal growth at all tested concentrations; therefore, the two oils were omitted in the rest of the investigation.

The enzymatic profile of *P. polonicum* showed marked activity of various hydrolytic enzymes, in the absence of the two oils, the fungus was able to produce nearly all cell-wall degrading enzymes (CWDE) that are known to be involved in the plant–pathogen interaction. These enzymes facilitate the infection route via the maceration of plant tissue.

The plant cell wall is a physical barrier against pathogenic invasion. Phytopathogenic fungi produce an array of CWDEs to invade host tissues through the degradation of cell wall components of plants [45]. Cell wall degrading enzymes are thought to help pathogens directly penetrate host tissues, and therefore, may be essential for pathogenicity.

The mode of action of every CWDE has already been reported. FPase (the overall cellulolytic activity of cellulase enzymes) works on cellulose that is widely distributed in plant cell walls. The hydrolysis of cellulose is accomplished by components of cellulase, including randomly acting endoglucanse, two exoglucanases (cellodextrinases and cellobiohydrolases), and β-glucosidase, that completely degrade cellulose into glucose units [20,46,47].

Xylanase (hemicellulose-degrading enzymes) manages the hydrolysis of xylan (the main part of the hemicellulose fraction). This process requires the combined action of various xylanases, including endo-1,4-β-xylanase; β-D-xylosidases; and acetylxylan esterase, resulting in the release of xylose monomers [21,48].

Pectin-degrading enzymes are another important group of CWDEs. Two major classes of pectinase (esterases and depolymerases) are known. PGase is the main kind of heterogeneous pectinase, which aids the hydrolysis of pectin polymers by cleaving the α-1,4-glycosidic bond, releasing galacturonic acid [23,49].

Amylase is a starch-degrading enzyme, the catalytic action of this enzyme (group β-amylase and γ-amylase) leads to the full hydrolysis of starch into simple monomers of glucose [50].

Inulinases and invertase act as β-fructosidases. Inulinases hydrolyze inulin to liberate fructose units in a single step from the non-reducing end, by the action of two main enzymes (endoinulinase—which is specific for inulin—and exoinulinase [51,52,53]). Invertases (β-fructofuranosidases) are a large group of glycoside hydrolases, which catalyze the hydrolysis of sucrose to an equimolar mixture of glucose and fructose—both inulinases. Invertase enzymes are active on sucrose and, more, possess the unique feature of being able to hydrolyze inulin [19,52].

Finally, the hydrolysis of nitrogenous compounds is mainly mediated by the action of several kinds of proteases that cleave protein into peptides and amino acids [54]. Depending upon the type of the reaction, proteases are sectioned into exopeptidases that catalyze the terminal peptide bond, and endopeptidases that cleave the non-terminal bonds between amino acids [55].

Interestingly, FPase, xylanase, and PGase were found in the filtrate of the pathogen, despite the composition of JA tubers lacking cellulose and xylan in their structure. The main reason explaining such data is based on the secretion nature of enzymes by the microorganism, which is either induced, constitutive, or both. The induced (inducible) enzyme is one whose secretion requires—or is stimulated by—a specific inducer (substrate). Whereas, the constitutive enzyme is on that is produced by a microorganism, regardless of the presence or absence of the specific substrate [52]. This leads to the conclusion that the current pathogen may have both mechanisms of enzyme secretion, representing an additional threat to JA tubers in storage.

Contrarily, the presence of an inducer substrate in the tuber components induces the synthesis of amylase, inulinase, invertase, and protease. These enzymes could also be constitutive. Such a complementary enzymatic system supports the observations of fungal survival under field and storage conditions. In addition to the enzymes described above, other accessory enzymes are required to cleave the linkage to side chains, i.e., side-chain cleaving enzymes, such as methyl esters and acetylation, or to split linkages to lignin [45].

As discussed earlier, different CWDEs, such as cellulases, hemicellulases, pectinases, and proteases work together to crack up the plant cell walls for the entrance of a pathogen, therefore, this group ultimately works to degrade the outer part of the JA tubers. On the other side, there are other enzymes (amylase, inulinase, and invertase) that work on the degradation of the cell components. This latter group is expected to catalyze the degradation of sugar contents of the tuber. JA tubers are rich in inulin and various other sugars that represent a suitable substrate for these enzymes. Additionally, the tubers have a wide array of vitamins, minerals, and amino acids [11], representing a nutritious medium that supports fungal growth and development during the pathogenesis process.

The current pathogenic *P. polonicum* has a complementary profile of hydrolytic enzymes, which is why *P. polonicum* causes a serious mold and completely deteriorates JA tubers under storage. Fortunately, this tight enzymatic capacity disappeared after the application of oils, with both oils showing a strong inhibition impact on both growth and enzymatic production systems. The enzymatic deactivation of essential oils is a mutual interaction between the microorganism, oil components, and concentration, for example, the eugenol content of clove oil as well as, the presence of an aromatic nucleus and phenolic OH-group can react with the target enzyme, resulting in the deactivation of enzymes, and alteration of the cell membrane [10]. This combined action leads to inhibition of all the biological processes of the microorganism.

During preservation, storage causes undesirable changes in the JA tubers. Storage usually results in high losses in quality, caused mainly by rotting, desiccation, sprouting, freezing, and inulin degradation. Concerning the disease severity, storage-fungi represent a serious problem, since plant infection takes place in the field and then extends to the storage [7,8], during which, the pathogen can grow on tubers that represent the natural preferred conditions for fungal growth. However, both oils succeeded in greatly minimizing the deleterious action of the pathogen, likely due to the possible modes of action discussed above. Regarding the defense-related phytochemicals, peroxidase is a key enzyme in defense-related processes, including the accumulation of lignin and phenolic compounds and suberization. The polyphenol oxidase enzyme catalyzes the oxidation of phenols to quinines, which are substances that are highly toxic to the pathogen, while phenolic compounds contribute to resistance through their antimicrobial properties against the pathogen. The increase in these oxidative enzymes and the phenol content is associated with the increase in plant resistance against fungal infection [10].

The structural features of JA tubers under storage showed marked variations. The presence of the pathogen caused a pronounced reduction in the dry matter in the infected control, accompanied by a gradual increment in sprouting, as well as weight loss. This principally suggests a loss of a certain quantity of water from the tuber. Moreover, during long-term exposure to storage conditions (without EOs application), shrinking and water losses were recorded in tubers.

Tuber sprouting occurred during storage when dormancy was broken and sprouting was activated, leading to an accelerated loss of water through the permeable surface of the sprout. This also leads to physiological aging of tubers, resulting in weight and quality losses [56].

During the current trial, peat moss was used which is traditionally used in food preservation because of its antimicrobial activity. This antimicrobial activity is possibly attributable to some of its bioactive components, such as sphagnan, a pectin-like polymer that inhibits microbial growth via electrostatic immobilization of extracellular enzymes and/or nitrogen deprivation, in addition to phenolics that inhibit the activity of extracellular enzymes of microbes, or other constituents such as sterols and polyacetylenes [57]. Peat moss has been approved as a storage medium; it has a relatively high-water retention capacity of up to 16–25 times its dry weight [58], providing a relatively low humidity around the tubers that blocks heat transfer within the peat moss, leading to decreases in water loss even during storage at 25 °C. However, dry matter content in JA tubers depends on many factors, such as storage conditions [59].

Pure carvone oil has a pronounced impact as a suppressor of potato tuber germination during storage. The main biomarker for the validity of potato tuber antioxidant enzyme activities (peroxidase and polyphenol oxidase) was detected [56]. Moreover, the sprouting inhibition by caraway oil was accompanied by significantly decreased weight loss [60]. The active compounds, limonene, and carvone, in caraway oil, are known to suppress the sprouting of potato tubers via the inhibition of mitochondrial respiration and reducing carbohydrate degradation sugars [61]. Carvone may play a more specific role in the sprout growth of potato tubers, such as by inhibiting the key enzyme in the mevalonate pathway, which is the main pathway of gibberellin biosynthesis [62].

Eugenol oil inhibits the eye sprouts by causing necrotic injury in the apical meristem of the bud, leading to sprouting necrosis [63]. Eugenol can reduce the rate of sprout growth during storage in non-dormant tubers. Additionally, eugenol showed the least number of sprouts and shortest sprouts, while eugenol can also reduce sugars in the treated tubers, a process that appears to occur due to lower consumption levels of soluble carbohydrates [64].

Carbohydrates, inulin, and protein contents varied during storage according to the treatments, both oils minimized the development of the deterioration process. A fresh JA tuber contains up to 80% water, 10.6–17.3% carbohydrates—mainly in the form of inulin (7–30%)—and about 2–8% protein [6,7,65]. However, these contents vary due to several reasons, such as variety, climatic changes, and storage conditions. For the long-term storage of JA tubers, there are various microbiological, enzymatic, and biochemical alterations, which may lead to tuber damage in carbohydrate and protein chemistry. However, the basic constituents of caraway oil (monoterpenes) tend to delay the breakdown of tuber components associated with the enzymatic system, as well as respiration and energy metabolic enzymes, maintaining the internal biochemical enzymatic activities at a minimum level [15].

## 5. Conclusions

The blue mold; *P. polinicum* was isolated and reported as a novel storage pathogen on JA tubers. The blue mold is caused by *Penicillium polonium*, having a complementary active hydrolytic enzyme profile, and is capable of destroying JA tuber components. The blue mold was isolated from the infected tubers as the first recorded case on JA tubers. It is already widely known that essential oils are cheap and safe. This provides economic benefits and if they are replaced with fungicides, then another value-added economic benefit will be gained. Therefore, this study suggests an economic oil-based conservation procedure to preserve JA from blue mold, and maintain the nutritive value of JA tubers when under storage conditions. Under room temperature, such tubers could be protected by using caraway and clove essential oils, in descending order. The tuber survived under infection stress for up to 4 months without a significant deterioration in quality characteristics being observed. This suggests the potential to implement this ecofriendly oil-based preservation procedure on a larger scale.

## Figures and Tables

**Table 1:** Chemical composition of essential oils from clove buds and caraway seeds.

Oil	Peak Number	Compound	Retention Time	Peak Area%	Chemical Structure of Major Oil
Clove	1	Methyl salicylate	6.928	19.274	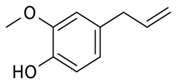 Eugenol
	2	Bicyclobutylidiene	7.645	3.084	
	3	Eugenol	11.080	49.077	
	4	Eugenyl acetate	11.603	11.551	
	5	Farnesene	11.957	3.324	
	*	Unidentified compounds	3.950	4.473	
	*		5.879	2.542	
	*		6.479	6.675	
	Total			100	
Caraway	1	Limonene	3.670	35.265	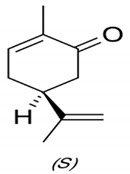 Carvone
	2	Carvone	9.469	60.398	
	3	Perilla alcohol	9.707	1.430	
	4	Carveol	2.088	1.106	
	*	Unidentified compounds	3.125	0.822	
	*		1.319	0.063	
	*		10.223	0.916	
	Total			100	

* means that this is not known “Unidentified compounds”.

**Table 2:** Enzymatic profile of the pathogenic *P. polonicum* in the presence of various levels of the tested oils (U mean ± SD).

Oil, %		FPase	Xylanase	PGase	Protease	Amylase	Inulinase	Invertase
0.0		17.62 ± 0.31	28.11 ± 1.04	38.71 ± 2.22	23.6 ± 3.33	56.6 ± 3.21	5.18 ± 1.81	0.93 ± 0.11
Clove	0.5	2.96 ± 0.22	23.28 ± 1.23	10.54 ± 1.94	23.59 ± 4.06	15.46 ± 2.34	4.92 ± 2.65	0.25 ± 0.11
	1.0	0.00	20.20 ± 0.97	8.74 ± 2.04	23.57 ± 2.55	11.59 ± 2.68	4.92 ± 1.54	0.24 ± 0.09
	1.5	0.00	16.25 ± 0.99	8.03 ± 1.55	21.63 ± 3.26	11.14 ± 1.45	4.87 ± 1.55	0.24 ± 0.08
	2.0	ND	ND	ND	ND	ND	ND	ND
	2.5	ND	ND	ND	ND	ND	ND	ND
Caraway	0.5	12.27 ± 0.44	0.00	18.97 ± 2.03	16.61 ± 3.52	32.96 ± 3.35	5.14 ± 0.62	0.27 ± 0.15
	1.0	8.30 ± 0.29	0.00	16.29 ± 1.05	15.00 ± 2.58	26.14 ± 2.56	5.00 ± 0.76	0.25 ± 0.13
	1.5	0.00	0.00	8.24 ± 2.21	13.76 ± 2.48	5.00 ± 1.02	4.95 ± 0.56	0.25 ± 0.09
	2.0	ND	ND	ND	ND	ND	ND	ND
	2.5	ND	ND	ND	ND	ND	ND	ND

ND: not determined due to full growth inhibition.

**Table 3:** Four months evaluation of decay severity (%) of JA tubers stored at 25 ± 2 °C and treated with essential oils under infection by *P. polonicum* (mean ± SD).

Treatment	Storage Time (Month)			
	1	2	3	4
C	18.1 ± 6.1	30.6 ± 3.5	57.9 ± 1.2	76.4 ± 1.9
P	48.3 ± 3.2	73.4 ± 5.1	ND	ND
CR	0.0	0.0	0.0	0.0
CL	0.0	0.0	0.0	0.0
CRP	0.0	0.0	0.0	0.0
CLP	9.5 ± 3.7	22.4 ± 3.8	45.5 ± 3.3	64.3 ± 3.5

C: untreated control; P: untreated infection by *P. polonicum* (ARS20); CR: treated with caraway oil; CL: treated with clove oil; CRP: treated with caraway oil plus infection by *P. polonicum*; CLP: treated with clove oil plus infection by *P. polonicum;* ND: not determined due to full deterioration.

## Data Availability

Relevant data applicable to this research are within the paper.
