# Correction: Al-Askar et al. Discovering *Penicillium polonicum* with High-Lytic Capacity on *Helianthus tuberosus* Tubers: Oil-Based Preservation for Mold Management. *Plants* 2021, *10*, 413

**DOI:** 10.3390/plants10091905

**Published:** 2021-09-14

**Authors:** Abdulaziz A. Al-Askar, Ehsan M. Rashad, Khalid M. Ghoneem, Ashraf A. Mostafa, Fatimah O. Al-Otibi, WesamEldin I. A. Saber

**Affiliations:** 1 Botany and Microbiology Department, Faculty of Science, King Saud University, Riyadh 11451, Saudi Arabia; ashraf812@yahoo.com (A.A.M.); falotibi@ksu.edu.sa (F.O.A.-O.); 2 Seed Pathology Research Department, Plant Pathology Research Institute, Agricultural Research Center (ID: 60019332), Giza 12112, Egypt; ehsanrashad78@yahoo.com (E.M.R.); khalid_ghoneem@yahoo.com (K.M.G.); 3 Microbial Activity Unit, Microbiology Department, Soils, Water and Environment Research Institute, Agricultural Research Center (ID: 60019332), Giza 12112, Egypt

The authors would like to advise that several corrections have been made to the original article [1]. The mistake in the spelling of the species name of *Penicillium polonicum* was corrected in the article, including the title and abstract.

Several of the scientific names were rewritten and italicized, and the abbreviation of the genus name of some others was added to replace the complete genus name.

In the first paragraph of the introduction, the name of the mold was written as blue or green mold, and several typos were corrected.

The authors apologize for any inconvenience caused and state that the scientific conclusions are unaffected. The original article has been updated.
